# Body–Brain Connections: The Effects of Obesity and Behavioral Interventions on Neurocognitive Aging

**DOI:** 10.3389/fnagi.2017.00115

**Published:** 2017-05-01

**Authors:** Chelsea M. Stillman, Andrea M. Weinstein, Anna L. Marsland, Peter J. Gianaros, Kirk I. Erickson

**Affiliations:** ^1^Department of Psychiatry, University of PittsburghPittsburgh, PA, USA; ^2^Department of Behavioral and Community and Health Sciences, University of PittsburghPittsburgh, PA, USA; ^3^Department of Psychology, University of PittsburghPittsburgh, PA, USA

**Keywords:** aging, brain health, intervention, obesity, physical activity

## Abstract

Obesity is a growing public health problem in the United States, particularly in middle-aged and older adults. Although the key factors leading to a population increase in body weight are still under investigation, there is evidence that certain behavioral interventions can mitigate the negative cognitive and brain (“neurocognitive”) health consequences of obesity. The two primary behaviors most often targeted for weight loss are caloric intake and physical activity. These behaviors might have independent, as well as overlapping/synergistic effects on neurocognitive health. To date obesity is often described independently from behavioral interventions in regards to neurocognitive outcomes, yet there is conceptual and mechanistic overlap between these constructs. This review summarizes evidence linking obesity and modifiable behaviors, such as physical activity and diet, with brain morphology (e.g., gray and white matter volume and integrity), brain function (e.g., functional activation and connectivity), and cognitive function across the adult lifespan. In particular, we review evidence bearing on the following question: *Are associations between obesity and brain health in aging adults modifiable by behavioral interventions?*

## Introduction

Obesity is an epidemic in the United States, affecting over one-third of American adults. Rates of obesity in middle aged (adults aged 45–64) and older adults (adults aged 65 and older) are amongst the highest of all age categories (Ogden et al., 2014) and are increasing. This upward trend is in sharp contrast to the declining or stable rates recently reported for other age categories (Ogden et al., 2016).

In addition to conferring risk for cardiovascular disease, recent evidence links obesity with unfavorable changes in brain morphology and function, as well as impairments in cognitive performance. When examined in older adults, these obesity-related effects go above-and-beyond the changes seen during normal aging. Whether associations between obesity, brain, and cognitive measures are a *consequence* of weight gain or are the *cause* of behaviors that subsequently lead to weight gain (i.e., hedonic overeating) remains a matter of speculation and rich debate (Schwartz and Porte, 2005; McEwen and Morrison, 2013). Nonetheless, there is now evidence for causal links between obesity and adverse changes in brain morphology and function. Accordingly, it is plausible that adulthood obesity may accelerate the onset of late-life neurocognitive impairments and disorders.

Although the factors leading to a population-wide increase in body weight are still under investigation (e.g., see Luke and Cooper, 2013; Blair, 2015; Malhotra et al., 2015 for debate), there is evidence that certain behaviors that reduce obesity could in turn improve aspects of neurocognitive health. The two primary behaviors most often targeted for promoting weight loss are diet (energy intake) and physical activity levels (energy expenditure). These behaviors might have independent or overlapping/synergistic effects on neurocognitive health.

Physical activity (PA) is a modifiable lifestyle factor shown to improve physical and neurocognitive health throughout the lifespan. PA has been associated with elevated cognitive performance and enhanced brain function in both neurologically healthy and impaired adults (Hughes and Ganguli, 2009; Bherer et al., 2013b; Gajewski and Falkenstein, 2016). For example, cross-sectional, prospective longitudinal, and randomized controlled trials have demonstrated that PA is associated with increased gray matter volume in the hippocampus and prefrontal cortex (Colcombe et al., 2006; Erickson et al., 2011; Ruscheweyh et al., 2011; Weinstein et al., 2012; Maass et al., 2015). These brain changes are sometimes associated with improvements in cognitive function, including memory and executive function (e.g., Erickson et al., 2011; Ruscheweyh et al., 2011; Weinstein et al., 2012; but see Young et al., 2015). Hence, the brain appears to have an enduring capacity for plasticity, and even modest changes in behavior can alter the size and functioning of the brain.

Prior reviews and meta-analyses have described the associations between obesity and behavioral interventions that promote neurocognitive health independently, and in parallel, from one another. This is striking since obesity and certain modifiable lifestyle behaviors (e.g., PA and energy intake) are closely linked both conceptually and physiologically. For example, obesity and PA levels are often correlated and predict many similar health outcomes (i.e., cardiovascular health). They also exert effects on similar physiological pathways and brain networks, as will be discussed further below. At the same time, there is also evidence that PA, diet, and obesity may influence different organ and disease endpoints, such as brain or cardiovascular health, through distinct pathways.

In this review, we first summarize evidence linking obesity, diet, and PA activity with brain morphology (e.g., gray and white matter volume and integrity) and function (e.g., functional activation and connectivity) across the adult lifespan. These sections are followed with a summary of the possible mechanisms of obesity and select behavioral interventions on neurocognitive health. In particular, we summarize evidence bearing on the following question: *Are associations between obesity and neurocognitive health in aging adults modifiable through behavioral intervention?*

## Effects of obesity on neurocognitive health

Obesity is a condition where there is an excess proportion of body fat in relation to lean mass, resulting from excess energy intake. Obesity status is typically assessed by measuring body mass index (BMI), which refers to the ratio of a person's weight to their height. According to the World Health Organization, a BMI of at least 30 kg/m^2^ is considered obese, between 25 and 30 is considered overweight, between 18.5 and 25 is considered healthy weight, and below 18.5 is considered underweight (National Institutes of Health, 1998). However, BMI is not actually a measure of body fat, but rather is a relatively crude measure used as a proxy for body composition. The correspondence between BMI and actual body composition varies by age and gender. For instance, even when holding BMI constant, healthy women and older adults tend to have more body fat than men and younger adults, respectively (Prentice and Jebb, 2001). Therefore, BMI is a rough, and sometimes poor, approximation of adipose vs. lean tissue. Other, more accurate measures of body fat exist (e.g., central adiposity metrics) and are more predictive of negative health outcomes (Sahakyan et al., 2015), but the methods for collecting these metrics are often more costly and/or time-intensive than calculating BMI. Thus, despite the well-known limitations of BMI, many of the studies described in this review have used BMI as the primary measure of obesity in adulthood.

### Obesity and brain health

#### Gray matter

Many studies have shown that obesity (typically assessed via BMI) is associated with reduced gray matter volume in both neurologically healthy and cognitively impaired older adults. This relationship has been noted consistently in the hippocampus (Ho et al., 2010a; Raji et al., 2010; Cherbuin et al., 2015). For example, Raji et al. (2010) compared the brain volumes of 94 obese, overweight, or healthy weight adults. Compared to healthy weight individuals, the obese adults had reduced gray matter volume in several brain regions, including the hippocampus, prefrontal cortex, and other subcortical regions. The group differences remained significant, even after controlling for comorbid conditions, such as diabetes, suggesting a unique role of excess body fat to brain atrophy. The hippocampus is of particular interest for considering the health trajectories of obese older adults because it is a region supporting episodic and relational memory functions. Moreover, this region is known to be particularly sensitive to pro-inflammatory proteins, metabolic disruptions, stress, and aging (Sudheimer et al., 2014). In fact, smaller hippocampal volumes are predictive of a future Alzheimer's Disease (AD) diagnosis in cognitively healthy older adults (e.g., Elias et al., 2000).

Obesity is also inversely related to the volume of gray matter regions in other brain areas. These patterns have been reported in a number of regions including the anterior cingulate and dorsolateral prefrontal cortex, orbitofrontal cortex, hypothalamus, basal ganglia, supplementary motor area, lateral occipital cortex, cerebellum, and brainstem (Jagust et al., 2005; Debette et al., 2010, 2014; Ho et al., 2010a; Raji et al., 2010; Walther et al., 2010; Brooks et al., 2013a; Medic et al., 2016). In one study, Medic et al. (2016) examined associations between BMI and cortical thickness in a sample of 203 adults (mean age 32 years) with no known physical or metabolic morbidities. There were no associations between BMI and global measures of brain health, such as average cortical thickness or total surface area. However, higher BMI was associated with reduced gray matter volume in a large cluster in the ventromedial prefrontal cortex, extending into the anterior cingulate and frontopolar cortex, as well as with a cluster in the lateral occipital cortex. The anterior frontal regions identified in this work support diverse behaviors such as reward and salience detection, autonomic nervous system control, decision making, and executive functioning. Posterior occipital areas are critical for functions such as visual attention. Thus, excess adiposity is associated with cortical thinning in brain regions underlying diverse cognitive functions, even in a sample free of the various comorbid health conditions associated with obesity. Of course, one caveat of the Medic study and others is that these studies are correlational and/or cross-sectional nature. The causal associations between brain health and obesity are difficult to determine because it is conceivable that degeneration of regions supporting visual attention and feeding behavior could be either a cause or consequence of overeating. Nonetheless, it is clear from this literature that obesity is consistently associated with lower gray matter volume in many cortical and subcortical brain areas.

Yet, not all studies have shown that obesity is associated with reduced brain volume. For example, Taki et al. (2008) reported both positive and negative correlations between BMI and gray matter volume in a sample of 1,428 middle-aged Japanese adults. BMI was negatively associated with total gray matter volume, as well as with volume in the medial temporal, occipital, and frontal lobes of older men (but not women). However, higher BMI was also associated with *greater* volume in other regions of the frontal, temporal, and cerebellar cortices in the same male sample. These findings are not consistent with the majority of other studies and could be explained by a number of factors, including differences across studies in sample size, analytical approach, or presence of comorbidities (e.g., diabetes).

#### White matter

There is mixed evidence regarding the direction of the associations between obesity and white matter volume. For example, while many studies find that greater BMI is associated with lower white matter volume in many subcortical and cortical regions (Ho et al., 2010a,b; Raji et al., 2010; Driscoll et al., 2012; van Bloemendaal et al., 2016), other studies fail to find a relationship between BMI and white matter volume (Gunstad et al., 2008; Soreca et al., 2009; Debette et al., 2010), and still others have reported a positive relationship (Pannacciulli et al., 2006).

Positive associations between obesity and white matter volume have been reported in the basal ganglia, frontal lobes, medial temporal lobe, precuneus, cerebellum, brainstem, and parts of the occipital cortex (Pannacciulli et al., 2006; Haltia et al., 2007; Walther et al., 2010). Although it is possible that these are merely spurious positive correlations, losing weight through dietary changes reduces white matter volume in similar regions (Haltia et al., 2007). For example, in a group of 30 obese and 16 lean middle-aged adults (mean age = 37 years), obese adults had greater white matter volume in the frontal lobes, fusiform gyrus, brainstem, and cerebellum than their lean peers. A subset of the obese group (*n* = 16) then underwent a 6-week, calorie restrictive diet that resulted in a reduction of white matter volume throughout some of the regions that had previously been larger in the obese compared to lean adults. These experimental data indicate that the positive relationships between white matter volume and adiposity in obese individuals may not be spurious since it can be experimentally reduced with only 6 weeks of dieting.

One possible explanation for the paradoxical reduction in white matter volume following caloric restriction is that morphometric analyses of white matter might be extracting information about local inflammation in addition to the integrity and volume of the axonal sheaths themselves. For example, both rodent and human studies find evidence of reactive gliosis in the hypothalamus, an area critical for body weight maintenance. In mice, a high fat diet induced hypothalamic inflammation both transiently and chronically, and obese humans exhibited increased gliosis, which was detectable on MRI images of the hypothalamus (Thaler et al., 2012). If morphometric measures of white matter volume are detecting glial cell density in the lipid-based myelin sheath, then a higher volume could indicate poorer white matter health (via more inflammation).

Diffusion tensor imaging (DTI) is a newer, more granular method of assessing white matter, which has led to a more consistent picture of obesity and white matter associations. The structural integrity of white matter tracts connecting various brain regions is generally reduced in obesity (e.g., Mueller et al., 2011; Verstynen et al., 2012; van Bloemendaal et al., 2016). Fractional Anisotropy (FA) is the most common measure of white matter integrity and indicates the shape of water diffusion in a given voxel. FA is calculated by taking the ratio of axial diffusivity (DA; along the axon) to radial diffusivity (DR; perpendicular to the axon). Lower FA can result from either a drop in DA (indicating less diffusion down the length of the axon) or an increase in DR (indicating more diffusion, or “leaking,” out of the axon).

Recent DTI work has shown that obesity-linked decreases in FA are due to both an increase in DR throughout the brain, as well as a decrease in DA (Mueller et al., 2011; Verstynen et al., 2012). For example, in a sample of 28 neurologically healthy adults (aged 18–69 years), Verstynen et al. (2012) demonstrated a consistent negative relationship between BMI and FA across diffuse WM pathways. Then, by controlling for overall water diffusion, they demonstrated that the decreases in FA were attributable to differences in both DA and DR (i.e., decreased DA and increased DR). Another study reported a similar association between increasing BMI and decreasing FA and, further, showed that increased DR accounted for a greater proportion of the difference in FA for females compared to males (Mueller et al., 2011). Across both sexes, obesity-related decreases in FA are most consistently observed in tracts connecting the frontal and temporal lobes, as well as those connecting limbic regions (for review see Kullmann et al., 2015). Interestingly, such a pattern of increased DR is also associated with demyelination in mouse models of inflammatory disorders, such as Multiple Sclerosis (Klawiter et al., 2011). Thus, the systemic increases in circulating inflammatory cytokines reported in obesity may contribute to the increases in DR signal (and resulting decreases in FA) observed in MRI studies of white matter integrity in obese adults (Song et al., 2005; Klawiter et al., 2011).

Demyelination also compromises the health of the remaining tissue within white matter tracts. White matter hyperintensities (WMH) are lesions in white matter, which are readily visible in MRI and could be a sign of demyelination processes. These lesions are observed more often in groups with ischemic and cardiovascular disease, conditions highly prevalent in obesity (Chimowitz et al., 1989). In addition, WMHs are positively associated with BMI (Jagust et al., 2005; Ho et al., 2010a) and visceral adiposity (Pasha et al., 2016) in cognitively normal middle aged and older adults. For example, in a sample of 126 middle aged adults (aged 40–62), Pasha et al. (2016) demonstrated across several objective measures of adiposity—e.g., waist circumference, and percent body fat—that higher body fat is associated with a greater WMH burden throughout the brain. Thus, multiple measures of both obesity and white matter provide converging evidence that excess adiposity is associated with poorer health and integrity of white matter. The fact that the participants in many of these studies are cognitively normal suggests that these associations may precede any detectable cognitive changes in these groups.

Together, these studies in aging adults suggest that obesity is associated with reduced health and integrity of gray and white matter in the brain. Given that normal aging is also associated with decreased gray and white matter integrity and increased WMH burden (Bennett et al., 2010; Raz et al., 2012), these findings suggest that obesity may accelerate the characteristic features of brain aging. However, some notable caveats of the studies described above are that they are predominantly cross-sectional in nature and have small-to-modest sample sizes. Thus, although they have provided provocative and somewhat consistent associations between obesity and brain health, they are ultimately limited in their ability to draw causal associations that are generalizable to larger populations. In particular, there are many conditions that often co-exist with obesity (e.g., diabetes, hypertension) that could be confounding or mediating these associations (e.g., Verstynen et al., 2013; Onyewuenyi et al., 2014). It is only with well-designed interventions or prospective longitudinal studies that the direction and temporality of these associations can be determined.

#### Functional brain activation

Functional neuroimaging studies comparing obese vs. healthy-weight individuals have identified obesity-related abnormalities in a wide range of brain areas. Most of these studies to date have been in younger and/or middle aged adults and have examined brain responses to food-related cues. In a systematic review, Pursey et al. (2014) reviewed 60 studies of obesity including functional magnetic resonance imaging (fMRI) outcomes. Compared to healthy-weight controls, obese adults consistently exhibit increased activation to food compared to non-food items in limbic and orbitofrontal brain regions associated with decision making, satiety, motivation, and reward (Pursey et al., 2014). While most studies have reported *hyper*activation of various brain regions (e.g., orbitofrontal cortex), there are also some regions in which *hypo*activation has been observed in a different set of reward-related (e.g., striatum) and cognitive control-related (e.g., dorsolateral prefrontal cortex) regions, suggesting general dysfunction in reward circuits in obesity with some regions showing greater and others less activation compared to healthy-weight individuals (Brooks et al., 2013b). Notably, functional activation differences between obese and healthy-weight groups are typically largest in response to high calorie food items, and have also been shown to scale with individual differences in BMI (Rothemund et al., 2007; Grosshans et al., 2012; Martens et al., 2013). This pattern suggests that obesity is associated with altered functional brain activation compared to healthy-weight counterparts, particularly in the context of food-related stimuli.

Consistent differences in brain activation of obese compared to healthy weight older adults have been reported as well, with some notable divergences from the pattern seen in younger groups. For example, Green et al. (2011) compared older and younger adults' functional responses to pleasant (sucrose) vs. aversive (caffeine) taste stimuli. In both age groups, BMI was negatively associated with activation of the caudate, a region of the striatum involved in motivation and reward during pleasant stimuli. However, in older adults BMI was also negatively associated with activation of the nucleus accumbens (NA), another central reward-related region. The NA is a key region initiating the reinforcing effects of addictive substances, such as drugs and highly palatable foods, as it receives dense innervation of dopamine producing neurons of the ventral tegmental area of the mesencephalon. Thus, the hypoactivation of certain reward-related regions in response to pleasant taste stimuli in obese older adults, coupled with the fact that dopamine declines in normal aging (Volkow et al., 2000), may indicate that older adults are particularly susceptible to food-related reward insensitivity and weight gain.

There is also evidence for aberrant brain function in obese adults, even outside of the context of food. In a fMRI study by Gonzales et al. (2010), cognitively normal obese and overweight middle-aged adults were scanned while completing a two-back working memory task in which participants indicated when a letter presented was the same as the one occurring two trials back. The authors then examined the functional activation across obese and non-obese groups in a set of *a priori* task-relevant regions. The obese group showed significantly lower activation in the right parietal cortex, a region involved in executive functioning, compared to the non-obese group, as well as worse performance on the working memory task. Further, both BMI and parietal activation covaried with individual differences in insulin sensitivity (assessed via blood draw by comparing levels of fasting glucose and insulin), and insulin insensitivity statistically mediated the relationship between BMI and parietal activation. Other groups have shown similar obesity-related hypoactivation using fMRI and peripheral markers of metabolic health and have also shown that obesity-related impairments in cognitive performance are linked to changes in neuronal viability and metabolism (Gonzales et al., 2010, 2012; Haley et al., 2013). These findings add to a growing body of literature demonstrating that obesity is associated with decreases in brain and cognitive health, which can be linked to mechanisms involving peripheral and central metabolic dysfunction.

### Obesity and cognitive function

As might be predicted given the negative links between obesity and the various aspects of brain morphology and function described above, obese adults generally perform more poorly on cognitive tasks compared to their healthy weight counterparts. Cognitive functioning in obesity is particularly impaired in the domains of executive functioning and memory. A recent review examined the relationship between obesity and cognitive function across a variety of cognitive domains during different developmental life stages (Smith et al., 2011). Across 15 cross-sectional and four prospective studies, in adults aged 19–65, greater body mass was associated with poorer performance on measures of global cognitive functioning, semantic and episodic memory, motor/processing speed, and, most consistently, executive function. Indeed, these studies were remarkably consistent, with 14 of the cross-sectional studies and three of the prospective studies showing negative associations. The remaining two studies were conducted in older adults aged 72 years or older and reported a positive association between BMI and cognitive functioning, which will be discussed further below.

There is also evidence that more time spent in an obese state has a cumulative negative effect on cognitive performance in adults (Sabia et al., 2009). A meta-analysis of 16 prospective studies concluded that midlife obesity, as defined by BMI, significantly increased the risk of developing dementia as compared to being normal weight in midlife (Anstey et al., 2011). Being underweight is also associated with dementia risk in late life (Beydoun et al., 2008). Maintenance of a healthy weight throughout adulthood (not simply *lower* weight) may therefore represent a critical component in preventing late life cognitive impairment.

### Mechanisms of obesity on neurocognitive health

The relationships between obesity and peripheral health can be linked to those found in the brain. In particular, excess adipose tissue has negative vascular and metabolic consequences on the body, including increased risk for atherosclerotic heart disease and Type II diabetes (Sharma and Chetty, 2005). Moreover, declines in vascular health lead to executive cognitive dysfunction (e.g., Forman et al., 2008), further supporting the potential role of vascular mechanisms in obesity-cognition associations. One major pathway by which obesity, especially central adiposity, can engender risk for heart disease is through associations with elevated blood pressure (e.g., Krauss et al., 1998). The relationship between heart disease and blood pressure is thought to occur because being overweight or obese is associated with activation of the renin-angiotensin-aldosterone system, elevated sympathetic nervous system activity, renal sodium retention, and increased levels of procoagulants in systemic circulation. These physiological changes can combine to increase cardiac output, endothelial dysfunction, and vessel stiffening (atherosclerosis and artiolosclerosis)—likely all consequences of the increased blood volume needed to maintain excess adipose tissue (e.g., Pausova, 2006).

Obesity can also lead to peripheral insulin resistance and hyperinsulemia, both conditions leading to increased circulating glucose levels. Importantly, insulin resistance and hyperinsulemia affect kidney function in a manner that increases sodium reabsorption and, consequently, blood pressure (Wickman and Kramer, 2013). An individual presenting with all of the above conditions—excess adiposity (particularly in central regions), elevated blood pressure, and glucose/lipid dysregulation—is considered to have metabolic syndrome (MeS; Grundy et al., 2004). Several studies have shown that the risk for MeS increases with age, suggesting that understanding its widespread health consequences in middle-aged and older adults will be important for future health care planning (Ford et al., 2002; Ravaglia et al., 2006; Hildrum et al., 2007). Most relevant to the present review, this particular clustering of diabetes mellitus and cardiovascular risk factors, referred to as MeS, has been linked to cognitive impairment, including dementia, as well as functional and structural changes in brain systems important for memory and higher order cognitive functions (Yaffe et al., 2003; Yaffe, 2007; Hassenstab et al., 2010; Yates et al., 2012).

Obesity has also been linked to central insulin resistance. That is, individuals with peripheral insulin resistance often also show reduced insulin sensitivity in the brain, and this can negatively impact cognitive functioning (Heni et al., 2015). Central insulin resistence has negative consequences on hippocampal-dependent and executive functions since the brain regions that support these functions (e.g., medial temporal and dorsolateral prefrontal cortices) have high concentrations of insulin receptors (Blázquez et al., 2014; Mainardi et al., 2015). Research in rodent models of obesity and Type II Diabetes support this idea, demonstrating that learning and memory functions are selectively impaired in insulin resistant rodents compard to healthy controls (Biessels and Reagan, 2015). In humans, regions essential for memory, including the hippocampus and dorsolateral prefrontal cortex, show reduced activation in obese compared to healthy-weight participants, and the degree of insulin resistance is negatively correlated with memory performance (Cheke et al., 2017). Together, the emerging research from both animals and humans converge on the idea that there are detrimental effects on brain insulin signaling as a result of obesity which are closely tied to peripheral metabolic dysfunction.

Compounding the negative effects of these metabolic conditions, excess adipose tissue secretes proteins, called adipokines, that can change the functioning of nearby tissues, signal the central nervous system, and ultimately have detrimental effects on brain health through inflammatory pathways (Mohamed-Ali et al., 1997; Xu et al., 2003). Circulating levels of inflammatory mediators covary positively with adiposity and systemic metabolic dysregulation (Ouchi et al., 2011). Converging animal and human evidence suggests that systemic inflammation plays a role in neurocognitive function by crossing the blood brain barrier and stimulating the production of central proinflammatory cytokines in discrete brain regions (Schöbitz et al., 1994; Vitkovic et al., 2000; Takeda et al., 2014). These central inflammatory mechanisms subsequently result in hippocampal neurodegeneration, impairment in memory function, and even heightened risk for dementia (Bellinger et al., 1995; Takeda et al., 2014). Further, cross-sectional evidence shows that among midlife adults, higher circulating markers of inflammation are associated with higher BMI, poorer working memory and executive function, and lower hippocampal volume (Marsland et al., 2006, 2008, 2015; Gianaros et al., 2015). Thus, peripheral inflammation—and interwoven metabolic dysregulation—is likely an important mechanism linking obesity to the neurocognitive outcomes discussed above.

### Interim summary

From the literature reviewed above it is clear that obesity is generally associated with reduced brain structure and function throughout the adult lifespan, and these associations likely have negative consequences for cognitive function. Physiological pathways, including inflammation, blood pressure, insulin resistance, and other cardiometabolic pathways, are also linked to brain structure and function. We therefore speculate that the effects of obesity on the more macroscopic aspects of brain and cognitive health may be mediated by these physiological systems. The critical question, however, is what can be done to reverse the negative consequences of obesity, or at least stall their progression.

Unfortunately, we still have a poor understanding of the broader behaviors driving the obesity epidemic and the interventions required to reverse this trend. For example, it is not clear to what extent obesity is attributable to decreases in energy expenditure (i.e., PA) vs. increases in caloric intake. This is a potentially important distinction as it might influence the effectiveness of certain types of interventions over others. In the following sections, we examine how PA interventions, as well as those that change body composition without targeting increased energy expenditure (e.g., dieting) may act through both overlapping and independent pathways to improve peripheral, brain, and cognitive health in obese adults.

## Effects of behavioral weight-loss interventions on neurocognitive health

If obesity is causally related to cognitive decline, then a reduction of body fat may attenuate obesity-related cognitive deficits. One well-known strategy to lose weight is via caloric restriction—i.e., dieting. The literature on diet-induced weight loss and cognition is still in its infancy, but preliminary evidence supports that weight-loss through caloric restriction can change aspects of cognitive functioning (Table 1). A meta-analysis examined seven randomized trials and five non-randomized studies of intentional weight loss in overweight and obese adults who were otherwise healthy (Siervo et al., 2011). While somewhat variable across studies, diet-induced weight loss improved attention and executive control. These effects were moderated by initial BMI, such that the strongest effects occurred in the most obese individuals, while overweight individuals showed no change in cognitive performance. Diet-induced weight loss also had a modest, marginally significant effect on memory function across the studies, but this trend disappeared when only studies including a control group were analyzed. While this meta-analysis examined the impact of diet-induced weight loss on cognitive performance, it is possible that dieting individuals also increased their PA levels in concert with healthier eating habits. Since PA is often prescribed in addition to diet for effective weight loss, it is unclear from these studies whether increased PA levels moderate (or mediate) fat loss-associated benefits on cognitive health.

**Table 1:** **Inervention studies showing effects of PA and/or caloric restriction on neurocognitive health in adults**.

**References**	**Type/Duration intervention**	**PA, Diet, or Both**	**Age group**	**Results**
Colcombe et al., 2004	6-month PA intervention	PA	Older adults	Greater activation in prefrontal, parietal, and anterior cingulate cortex, and well as increased cognitive control performance in higher aerobically fit (study 1) or aerobically trained (study 2) individuals.
Colcombe et al., 2006	6-month aerobic exercise trial	PA	Older adults	Aerobic exercise increased gray matter in the right middle frontal gyrus, left superior temporal lobe, and bilateral regions of the anterior cingulate and supplementary motor area, as well as in anterior white matter tracts.
Erickson et al., 2011	1-year aerobic walking intervention	PA	Older adults	Volume of the anterior hippocampus and memory performance increased following a 12-month aerobic exercise intervention.
Espeland et al., 2016	10-year “lifestyle” intervention involving PA and diet	Both	Older adults	Mean white matter hyperintensity volume was 28% lower among lifestyle intervention participants compared with those receiving diabetes support and education. The lifstyle intervention group also exhbited smaller ventricle volume compared to the education control group.
Haltia et al., 2007	6-week dietary restriction	Diet	Young-middle aged adults	White matter volumes were greater in obese compared to lean subjects in several basal brain regions. Positive correlation between white matter volume in basal brain structures and waist to hip ratio in obese individuals. The detected white matter expansion was partially reversed by dieting. Regional gray matter volumes did not differ significantly in obese and lean subjects, and dieting did not affect gray matter.
Honea et al., 2016	3-month intervention involving caloric restriction, increased PA, and behavioral modification	Both	Middle aged adults	Percent weight loss was positively correlated with baseline gray matter volume in right parahippocampal and orbitofrontal gyri in sccessful obese dieters. Successful dieters showed greater volume loss in the left precentral gyrus and the insula compared with unsuccessful dieters. A negative correlation was found between weight loss and pre-post-intervention volume change in left prefrontal regions.
Jakobsdottir et al., 2016	8 week diet (2 week isocaloric, 4 weeks caloric retriction, 2 weeks isocaloric); prospective intervention (no control)	Diet	Older adults	Activity in the amygdala and correlations between peripheral metabolic factors with activity in brain areas involved in food reward processing differ substantially before and after weight loss.
Krogh et al., 2014	3-month aerobic PA intervention	PA	Older adults	No Group × Time interactions (potentially due to poor adherence in intervention group). However, positive association between change in hippocampal volume and changes in memory.
Legget et al., 2016	6-month aerobic walking program	PA	Obese Young-middle aged adults	PA-related changes in communication between large-scale brain networks and integrative netowrk “hub” regions.
Maass et al., 2015	3-month aerobic PA intervention	PA	Older adults	Hippocampal volume, perfusion, and memory increases following a 3-month aerobic exercise intervention; Changes in perfusion statistically mediate relationship between volume and memory.
McFadden et al., 2013	6-month aerobic exercise intervention	PA	Middle aged adults	The intervention was associated with a reduction in DMN activity in the precuneus, which was associated with greater fat mass loss.
Niemann et al., 2014	12-month PA intervention	PA	Older adults	Increased hippocampal volume in both PA and control (coordinative training) groups.
Pajonk et al., 2010	3-month aerobic PA intervention	PA	Young-middle aged adults (schizophrenic patients)	Hippocampal volume and short-term memory improve following 3-month aerobic exercise intervention; Changes in volume correlate with changes in memory.
Paolini et al., 2015	6-month intensive weight loss RCT	Diet	Middle-older adults	After accounting for known correlates of weight loss (e.g., baseline weight, age, sex, and self-regulatory efficacy), greater global effiency in the HBN-A was associated with an additional 19% of the variance in weight loss.
Prehn et al., 2017	12-week caloric restriction	Diet	Older adults	In the caloric restriction group, found improved recognition memory, paralleled by increased gray matter volume in inferior frontal gyrus and hippocampus, and augmented hippocampal resting-state functional connectivity to parietal areas.
Rosano et al., 2010	12-month PA intervention	PA	Older adults	2 years after conclusion of intervention, PA group showed higher processing speed scores and significantly greater activation in processing-speed-related brain regions.
Rosano et al., 2016	24-month PA intervention	PA	Older adults	Greater hippocampal volume in PA compared to control group following moderate intensity exercise intervention, even after controlling for dementia-related covariates.
Ruscheweyh et al., 2011	6-month PA intervention	PA	Older adults	Positive association between changes in PA and episodic memory following a 6-month mixed-intensity intervention no longer significant after accounting for the variance associated with change in anterior cingulate gray matter volume.
ten Brinke et al., 2015	6-month aerobic exercise intervention	PA	Older adults	Hippocampal volume increases following 6-month aerobic exercise intervention. Changes in volume negatively associated with changes in short-term memory.
Voss et al., 2010	6-month aerobic PA intervention	PA	Older adults	Connectivity of large-scale brain networks and executive functioning improve following 6-month aerobic exercise intervention; changes in connectivity correlate with changes in executive functioning.
Voss et al., 2013	1-year aerobic walking intervention	PA	Older adults	Overall, no significant white matter changes, but percent change in VO2 positively predicted percent change in white matter FA in prefrontal, parietal, and temporal regions in the exercise group; FA did not relate to cognition.
Voss et al., 2013	12-month PA intervention	PA	Older adults	Improved fitness is associated with changes in prefrontal and temporal white matter integrity following a 12-month aerobic exercise intervention; no changes in short-term memory.

*For brevity, only interventions including brain outcomes are included; numerous interventions have shown cognitive (only) outcomes. FA, fractional anisotropy; PA, physical activity; V0_2_, maximal volume oxygen consumption*.

Although limited, there is also evidence that dieting may change brain structure and functioning. Jakobsdottir et al. (2016) conducted a prospective dietary intervention in 18 obese, but otherwise healthy, post-menopausal women (mean age 57 years; mean BMI = 32.2 kg/m^2^). The women were placed on a calorie-restrictive diet for 4 weeks and lost an average of 4.8% of their body weight. Participants completed a visual food cue task during functional magnetic resonance imaging (fMRI) before and after the caloric restriction intervention while in a fasting state. Compared to the baseline scan, following the intervention women showed less activation in response to food cues in some brain regions supporting motivation, salience and reward processing (e.g., amygdala) and more activation in other brain regions, including those supporting cognitive control (e.g., prefrontal cortex), suggesting that the functioning of cognitive control networks increases following dietary restriction. Since the amygdala is involved in motivational functions such as food craving, the authors suggested that the intervention modified signaling of satiety, perhaps via increased cognitive control signaling. These activation changes were also correlated with reductions in peripheral metabolic health markers (e.g., inflammatory cytokines, leptin), supporting the potential mediating role of peripheral systems on brain health. Unfortunately, while obesity-related comorbidities were controlled for (i.e., because all obese participants were otherwise physically healthy at baseline) participants' PA levels were not assessed, so it is possible that changes in PA may contribute to the reported effects (e.g., if participants also increased their PA during the intervention). Further, the existing study had a very small sample size (*N* = 18). Thus, there was not sufficient power to examine the potential mediating or moderating role of the peripheral metabolic biomarkers assessed. Despite these limitations, the findings reported in this initial interventional study support that diet-induced weight loss increases functioning in brain regions underlying executive control and decreases those implicated in food-related reward responses. Cross-sectional studies comparing obese to normal weight control groups support this conclusion, showing similar patterns of increased activation (McCaffery et al., 2009) and increased gray matter volume (Hassenstab et al., 2012) in cognitive control networks in healthy weight compared to obese groups. Given the paucity of studies in this area, future research is needed in order to further clarify the complex relationships between adiposity, cognitive control, and reward.

Interestingly, brain morphometry in cognitive control-related regions also increases following weight *gain* in individuals recovering from anorexia nervosa, a condition in which individuals are severely underweight (Seitz et al., 2013, 2016). This work serves to further bolster findings from dietary weight loss interventions in obesity because it suggests that the brain morphology differences observed across normal and overweight/underweight individuals are linked to unhealthy (either too high or too low) levels of adiposity rather than to another, unknown variable covarying with weight status.

### Mechanisms of caloric restriction on neurocognitive health

Although the mechanisms by which caloric restriction leads to changes in neurocognitive health in humans are not well-understood, it has become increasingly clear that proteins involved in metabolic regulation are likely key mediators of diet-induced neurocogniitve plasticity. This is because during an energetic challenge, the body switches from using liver glycogen to using adipose-derived fatty acids (Longo and Mattson, 2014). A host of metabolic benefits arise from this switch. For example, insulin sensitivity in the hippocampus increases following a period of caloric restriction (with or without exercise) in obese young and middle-aged adults (Larson-Meyer et al., 2006; Weickert, 2012), and intermittent fasting improves cardiovascular and hormonal responses to stress in rats (Wan et al., 2003). Fasting has also been shown to improve learning and memory consolidation in rodent models (Fontán-Lozano et al., 2007). However, more research in both animals and humans is needed to clarify the underlying cellular and molecular mechanisms of caloric restriction on brain health outcomes.

### Interim summary

Although limited, there is evidence that dietary interventions can improve cognition and brain functioning in areas that overlap with those showing associations with obesity. This pattern of results suggests that PA may not be a necessary component to interventions designed to reverse obesity-related neurocognitive dysfunction. However, none of the studies of dietary interventions to date have controlled for PA levels, and obesity-related health comorbidities have not been consistently accounted for, making it impossible to determine whether the effects attributed to diet are moderated or mediated by PA or other health-related factors. This possibility needs to be examined in the context of future dietary intervention studies.

In addition to dietary interventions, there are other ways to change body composition. PA is commonly prescribed alongside weight loss interventions for effective weight management. It can be difficult to disentangle the overlapping vs. independent effects of PA compared to other weight-loss strategies on neurocognitive health. However, doing so is important for increasing our understanding of which intervention components are most critical for promoting and preserving brain health.

## Effects of physical activity on neurocognitive health

As shown in Table 1, the majority of behavioral intervention research to date has focused on modifying physical activity (PA) levels to preserve neurocognitive health in older adulthood. PA is an umbrella term for bodily movement requiring energy (Caspersen et al., 1985). PA interventions are particularly attractive for promoting and preserving brain health in adulthood as they are low cost and widely accessible. PA has even been prescribed by some as the key ingredient for reversing the obesity trend, inadvertently perpetuating a common public misconception that you can “out run” poor dietary behaviors (Donnelly et al., 2009). However, there is also evidence that PA is not particularly effective compared to other types of interventions (e.g., dieting) at decreasing weight, especially in the dosage included in most intervention studies (e.g., see Wing, 1999; Donnelly et al., 2009). Instead, PA interventions more reliably change body composition (i.e., increase lean muscle mass and decrease adiposity), which can help prevent weight gain (Jakicic et al., 2008; Jakicic, 2009). These characteristics make PA interventions an interesting test bed in which to tease apart the key ingredients of interventions designed to mitigate the neurocognitive consequences of obesity.

In typical aerobic PA interventions, relatively inactive adults (typically older adults) are assigned to either an aerobic PA group or non-aerobic control group. Cognitive and brain functioning is assessed prior to and following the intervention period, which lasts 6–12 months in most PA studies. We review the evidence from aerobic PA interventions in the following sections and compare these effects, when possible, to the effects of intentional energy restriction (i.e., dieting). By doing this, we aim to get a better sense of the effects unique to aerobic PA (hereafter referred to as PA) vs. those that can be achieved from other interventions that change body composition without influencing cardiovascular health status.

### PA and brain health

#### Gray matter

Intervention studies that randomly assign adults to either receive PA or a control condition find that increased engagement in PA can alter the size and function of several different brain areas. For example, in one study, 59 older adults were randomly assigned to either a moderate intensity walking group or to a stretching and toning control group for 6-months (Colcombe et al., 2006). After the intervention, the aerobic exercise group showed increased gray matter volume in the frontal cortex (anterior cingulate cortex, middle frontal gyrus, and supplementary motor area), as well as in anterior callosal white matter tracts (also see Ruscheweyh et al., 2011). In contrast, the control group showed volumetric decreases that were consistent with normal aging. Similar effects were also found for a 1-year exercise intervention when examining the size of the hippocampus. In a sample of healthy older adults (mean age ~66 years), walkers showed a 2% increase in hippocampal volume as compared to their stretching and toning peers, who lost ~1.4% of their hippocampal volumes over this same period (Erickson et al., 2011). These exercise effects were relatively specific to the hippocampus in that the caudate nucleus and thalamus volumes were unaffected by the aerobic exercise intervention. A similar increase in hippocampal volume was recently replicated in a sample of 86 healthy female older adults following a 6-month aerobic PA intervention (ten Brinke et al., 2015), as well as following a similar 24-month intervention in 26 older adults with impaired mobility and cardiovascular health conditions (Rosano et al., 2016). Increases in hippocampal volume were also observed in a separate sample of 96 older adults following 12-months of aerobic or coordination training (Niemann et al., 2014). Thus, in as little as 6-months, PA can increase regional gray matter volume in adults, especially in the hippocampus and frontal cortex. Although not the focus of the present review, results of cross-sectional and correlational studies relating gray matter volume to PA and/or cardiovascular fitness generally support the findings from randomized PA interventions (e.g., see Colcombe et al., 2006; Hillman et al., 2008; Hayes et al., 2013; Erickson et al., 2014). Therefore, PA appears to be a promising method for increasing gray matter volume in adulthood, particularly in regions in which obesity and aging are associated with decreased gray matter volume.

#### White matter

White matter volume and integrity have been less often studied as outcome variables from randomized PA interventions. However, recent evidence supports that white matter integrity increases following experimental increases in cardiovascular fitness. In the first DTI study to examine changes in white matter integrity in the context of a PA intervention, Voss et al. (2013) evaluated the effects of a 12-month aerobic exercise program in 70 healthy older adults in which hippocampal gray matter had been shown to increase post-training (Erickson et al., 2011). Participants were randomly assigned to either an aerobic walking or toning/stretching (control) group and participated in their respective activities for 40 min per day, 3 days per week. Intervention-induced changes in cardiovascular fitness were associated with increases in FA in tracts connecting temporal and prefrontal brain regions. Although interventional evidence of PA-induced changes in white matter integrity is limited, there is a wealth of cross-sectional and correlational evidence supporting a link between these two variables (e.g., see Erickson and Kramer, 2009; Bherer et al., 2013a; Oberlin et al., 2016). Thus, as with the gray matter findings above, the interventional and cross-sectional findings converge on the idea that white matter integrity may improve with PA and increasing fitness.

There is also evidence that PA interventions can decrease the progression of WMH burden in adults. In a recent study, Espeland et al. (2016) evaluated the effects of a 10-year weight loss intervention involving both diet and physical activity on WMHs in a subset of 319 older adults from the Look Ahead trial, a randomized controlled trial of PA in older adults with Type II diabetes. All participants were overweight or obese and had diabetes at enrollment. At year 10, mean WMH volume was 28% lower in older adults who had participated in the intervention compared to a control group who had received educational classes on the same topics. These findings suggest that a long-term intervention involving PA may delay the progression of WMH burden associated with obesity and comorbid conditions, such as diabetes. However, it is not possible to disentangle the contribution of PA from those of diet in the effects observed, as the intervention involved both PA and nutritional counseling. Similarly, it is not possible to link *change* in WMH burden to PA, as baseline WMH measurements were not available. Nonetheless, the randomized controlled design of this study makes it less likely that the group difference in WMH burden observed were due to preexisting differences between the intervention and control groups.

In the case of WMH, cross-sectional and longitudinal studies of WMH do not provide any additional clarity regarding whether PA slows their progression in the brain. A recent meta-analysis examined the association between physical activity and WMH in individuals without advanced disease associated with WMH, such as stroke, depression, and dementia (Torres et al., 2015). Across 12 studies using WMH as an outcome variable, 6 studies reported that more PA was associated with less WMH burden in older adults, while the other 6 reported no association. The studies were more likely to find an association between PA and WMH if they used a longitudinal design, took PA across the lifespan into consideration (i.e. as opposed to only assessing PA at one time point), used a slightly younger older adult sample (mean age 68.5 vs. 72.8), used objective measures of PA (i.e., as opposed to self report), and performed multivariate analyses to control for risk factors associated with WMH. Thus, the null effects found in some studies examining the relationship between PA and WMH could be the result of design or analytical limitations, rather than a true null effect. Further, few PA studies have taken potential confounders, such as BMI and/or adiposity or obesity-related comorbid conditions into consideration. More research is therefore needed in this area before firm conclusions about a causal relationship between PA and WMH, and whether PA can reduce WMH burden in the same regions in which WMHs increase with obesity.

#### Functional brain activation

PA-related changes in brain function have been examined in the context of several PA interventions (e.g., Voss et al., 2010; Kamijo et al., 2011; Chaddock-Heyman et al., 2013; Hillman et al., 2014; Krafft et al., 2014). Unlike randomized intervention studies examining structural outcomes, most RCTs including functional outcomes have focused on changes in prefrontal cortex functioning rather than in the hippocampus. For example, 2 years after completing a 12-month aerobic PA intervention, a subsample of older adults who had continued to adhere to the intervention showed increased activation in bilateral prefrontal regions supporting executive control compared to those who has been in the control group (Rosano et al., 2010). Since bilateral activation in older adults could be compensatory (e.g., Cabeza, 2002), these results suggest that PA may lead to behaviorally relevant changes in the allocation of neural resources. These findings also suggest that the functional brain changes observed following PA interventions are sustained, even years after the termination of the intervention.

Functional connectivity between brain regions has also been shown to change in adults following PA interventions. Using fMRI, Voss et al. (2010) showed that a 12-month walking intervention increased functional connectivity among regions within two large-scale brain networks in older adults: The default mode and the frontal executive networks (FEN). The increased functional connectivity in the FEN, a network that includes several prefrontal brain regions, was associated with improvements in executive control performance. A seminal study by Colcombe et al. (2004) reported similar findings in the functioning and recruitment of the FEN following a shorter, 6-month exercise intervention in older adults, supporting the claim that changes to large scale brain networks may occur relatively soon after the commencement of exercise training. Since large-scale brain networks are known to become less efficient and less flexible with age, these results suggest that exercise may exert more global effects on the efficiency and flexibility in which networks of brain regions interact in older adults, leading to preserved cognitive performance.

### PA and cognitive function

The effects of PA on cognitive functioning are summarized extensively elsewhere (e.g., Cotman and Berchtold, 2007; Kramer and Erickson, 2007; Laitman and John, 2015), and so we only provide a summary of the cognitive effects here. The majority of research on PA and cognition focuses on whether this behavior can improve late-life, as opposed to midlife, cognitive performance. In a meta-analysis of 18 randomized PA interventions with older adults (ages 55–80), those involving aerobic PA training showed significant improvements on tasks of visuospatial processing, learning and memory, processing speed, and executive function when compared to non-aerobic control groups (Colcombe and Kramer, 2003).

While many domains of cognitive performance have been shown to improve with PA exposure, executive function tasks typically exhibit the largest and most consistent benefits (Colcombe and Kramer, 2003; Deary et al., 2006). Executive control is typically the first cognitive domain to show age-related decline and is also a cognitive domain in which obese adults are especially impaired. Therefore, the fact that executive functions are especially sensitive to the effects of PA suggests that PA interventions may be able to preserve and even enhance functioning in cognitive domains that exhibit impairments in both obesity and aging. This might make PA interventions particularly suitable for altering the course of brain function in obese adults. However, not all studies have reported significant changes in cognitive functioning following PA interventions (e.g., Sink et al., 2015; Young et al., 2015), thus the critical components of successful PA interventions (e.g., dosage, frequency, and duration) are still unknown.

### Mechanisms of PA on neurocognitive health

As with obesity, to understand the effects of PA on the more macroscopic neurocognitive outcomes discussed above, we again turn to peripheral processes. Regular PA confers protection against cardiovascular disease, Type 2 diabetes mellitus, and hypertension (e.g., Espeland, 2007; Jackson et al., 2014; Soares-Miranda et al., 2016). As mentioned above, cardiometabolic disorders are also associated with chronic systemic inflammation, as well as peripheral and central insulin resistance. One proposed mechanism of PA on the brain is through the reduction in systemic inflammation. PA interventions reduce pro-inflammatory and increase anti-inflammatory cytokines, and they may help to protect against chronic low-grade systemic inflammation (Petersen and Pedersen, 2005). This later point is especially important because levels of systemic inflammation increase in normal age-related cognitive decline and incident dementia (Engelhart et al., 2004; Rafnsson et al., 2007; Marioni et al., 2009). Specifically, cognitive dysfunction, especially Alzheimer's disease (AD), is associated with elevated blood levels of pro-inflammatory markers, such as C-reactive protein and interleukin-6 (e.g., Ravaglia et al., 2007). Rodent models offer a potential mechanism for the PA-dementia relationship in that reducing peripheral inflammation through PA-related decreases in amyloid-beta deposits in the brain, a hallmark of AD pathology and a putative cause of dementia (Nichol et al., 2008). Additional work has found that PA may also increase peripheral amyloid clearance mechanisms, suggesting that it may increase the overall ability of the body to regulate levels of proteins associated with neuropathology (Stranahan et al., 2012).

Other possible pathways by which PA improves neurocogntinve function involve increasing insulin and neurotrophic factor signaling in both the brain and periphery. In regards to insulin signaling, middle aged adults with higher levels of objectively measured PA have been shown to have higher insulin sensitivity compared to those with lower PA levels (Balkau et al., 2008), and those with greater insulin sensitivity exhibit better memory and more activation in memory-related brain networks than those with lower sensitivity (Cheke et al., 2017). Regarding neurotrophic factor signaling, PA interventions in rodent models have consistently shown increased levels of peripheral and central neurotrophins (e.g., brain derived neurotrophic factor) following periods of increased PA (e.g., Cotman et al., 2007; van Praag et al., 2014). However, measuring levels of neurotrohic factors in humans following PA is much more difficult. As a consequence, far less evidence on PA and neurotrophic factor signaling in humans exisits, although there is indirect evidence that neurotropic signaling may mediate the positive effects of PA on neurocogntion (Huang et al., 2014; Leckie et al., 2014; Mueller et al., 2015).

Together, the mechanistic evidence described above suggests that increased PA influences similar physiological pathways (i.e., inflammation, vascular health, metabolic signaling) as that of obesity, albeit in the opposite direction. Such patterns could suggest mechanisms by which changes in body composition through an increase in PA influences brain health.

### Interim summary

PA interventions involving even moderate-vigorous levels of PA are not particularly effective at reducing body weight. Rather, these interventions change cardiovascular fitness levels, as well as the body's composition of fat to lean muscle mass. Improvements in neurocognitive health observed following PA interventions in obese adults typically depend on changes in cardiovascular fitness rather than in weight loss or body composition *per se*. This suggests that weight and adiposity are not the most critical components for PA-related neurocognitive improvements.

Complicating this conclusion, however, is the fact that dietary interventions do not improve cardiovascular fitness nor increase lean muscle mass, yet improvements in neurocognitive health are still observed following these interventions. Although the evidence is limited, the effects of dieting appear to overlap with, but may be more selective than, the effects of PA interventions. This pattern highlights a potential unique role of cardiovascular fitness to brain and cognitive heath. A key caveat of the existing literature is that measures of BMI or adiposity are rarely included as covariates in PA studies. Conversely, PA is rarely included as a covariate in dietary restriction studies. This limits the causal links that can be drawn between these interventions and improvements in neurocognitive health.

## Discussion

Both aging and obesity contribute to increased health care costs. Consequently, an increase in the proportion of middle-aged and older adults who are obese may predict a compounded health care spending crisis in the future. Finding cost-effective, non-pharmacological interventions to attenuate the negative consequences of obesity on neurocognitive health is therefore a major public health priority. Until now, research examining the association between obesity and neurocognitive function has been mostly discussed separately from results relating PA and fitness to neurocognitive function. As described in the introduction, although PA and obesity are not simply inverse constructs of one another, there are clear physiological and conceptual similarities between them. Hence, this review brought these two literatures together to examine whether associations between brain and obesity are similar to those between brain and PA.

### Convergence and divergence in the effects of obesity vs. PA

PA interventions have been shown to increase neurocognitive health in similar areas as those affected by obesity and aging. In terms of brain health, the effects of PA are especially robust in the hippocampus and prefrontal cortex, as well as the white matter tracts connecting these regions. In the cognitive realm, PA interventions exert the largest and most consistent effects on executive control, while some less consistent effects are also observed in learning and memory processes. In addition, at the physiological level, there is evidence that PA works through similar mechanisms, albeit in the reverse direction, as obesity. For example, obesity is associated with increased peripheral inflammation and insulin resistance, as well as increased risk for cardiovascular (e.g., hypertension, diabetes) and neurodegenerative disease (e.g., dementia). Conversely, PA interventions decrease inflammation, increase insulin sensitivity, and decrease risk for a similar set of diseases (e.g., see Ertek and Cicero, 2012), suggesting that the mechanisms underlying obesity and PA converge. Such an overlap in underlying mechanisms could help explain the overlap in brain regions and cognitive functions most strongly linked to obesity and PA.

There are also some areas in which the effects of obesity and PA diverge. For example, as reviewed above, obesity has consistently been shown to affect the structure and functioning of limbic and reward-related brain networks, including regions such as the striatum and nucleus accumbens. On the other hand, PA studies have not shown consistent effects in the striatum or in limbic regions other than the anterior cingulate cortex. Therefore, the effects of obesity and PA on brain outcomes may not be merely opposites of each other. Further supporting this point, interventions (e.g., dietary interventions) that combat obesity by decreasing body fat composition, but do not also increase fitness levels or lean muscle mass show more selective improvements in cognitive performance (e.g., increases in episodic memory, but not executive functioning) and brain health (e.g., diffuse increases in white matter, but only select increases in gray matter structure) compared to PA interventions. This pattern suggests that there are unique benefits of PA interventions on neurocognitive health that are not necessarily attributable to changes in body composition. Similarly, there may be unique negative consequences of obesity that are not attributable to physical inactivity, such as altered reward sensitivity and responses to food-related stimuli.

### Converging cellular and molecular mechanisms

At the cellular and molecular level, several mechanisms emerge that could explain the pathways for obesity on brain function, while PA and energy restriction positively impact, neurocognition during aging. We do not go into great detail on these mechanisms here as they are summarized extensively in numerous reviews (e.g., Cotman et al., 2007; Neufer et al., 2015; Stranahan, 2015; Raefsky and Mattson, 2017). However, some commen themes are worth reiterating. First, obesity decreases metabolic function (e.g., impairs insulin signaling, decreases mitochondrial efficiency), increases glutocorticoid and inflammatory signaling, and decreases structural and functional integrity of brain circuits (most consistently in medial temporal and frontal lobes). As a consequence of these deleterious cellular and molecular effects, obesity is associated with deficits in cognitive functioning.

Fortunately, there is a wealth of evidence that behavioral interventions, such as PA or caloric restriction, work in opposition to obesity in each of the mechanistic cellular/molecular domains mentioned above (Neufer et al., 2015; Raefsky and Mattson, 2017). For example, PA improves metabolic function in both brain and periphery by increasing insulin sensitivity and mitochondrial biogenesis/efficiency. PA also decreases glutocorticoid secretion and central and peripheral inflammation (e.g., for review see Neufer et al., 2015). Finally, PA selectively increases neural plasticity (e.g., by increasing brain-plasticity-promoting neurotrophins) and cognitive functioning in domains similar to those affected in obesity, including memory and learning and executive control (e.g., Erickson and Kramer, 2009).

Caloric restriction has also been found to have positive effects on the cellular and molecular level (Longo and Mattson, 2014; Raefsky and Mattson, 2017). For example, dietary restriction or intermittent fasting increases mitochondrial biogenesis and efficiency, reduces oxidative stress, and enhances neurogenesis and the synaptic plasticity of neurons. These findings suggest that bioenergetically challenging the body, either through PA or through dieting/fasting have positive consequences at the cellular/molecular level which promote a healthier body and brain. These interventions hold considerable promise for reversing the negative consequences of obesity and associated metabolic diseases.

### Optimizing obesity interventions

A critical point of debate arising from these literatures is whether fatness or fitness matters more in terms of preserving neurocognitive health in adulthood. The answer to this question could inform the design of interventions for obesity in order to optimize neurocognitive health outcomes. Speaking to this point, the salutary effects of PA can be dissociated from changes in body composition in several ways. For example, PA can attenuate disease risk in individuals with varying levels of body fat, ranging from lean to obese (Church et al., 2005; Lee et al., 2005). Relatedly, neurocognitive changes have been observed following PA interventions, even in the absence of weight loss (Erickson et al., 2011; e.g., Erickson and Kramer, 2009). For example, following a 12-month aerobic exercise intervention, Erickson et al. (2011) reported improvements in spatial memory, as well as in hippocampal volume in a sample of cognitively normal older adults. These changes were associated with changes in fitness, but not with changes in body weight. This evidence again supports the notion that the mechanisms underlying PA and obesity are not just opposites of one another. That is, weight loss may not be a *necessary* component to mitigate the negative neurocognitive consequences of obesity. This signifies a potentially unique contribution of PA-induced changes in cardiovascular fitness (and not necessarily body composition) to the peripheral mechanisms discussed above. If this is the case, the associations between obesity and brain outcomes reported in the obesity studies above may not in fact be causal. Rather, they could be at least partially attributable to obesity-related health comorbidities, or to poor cardiovascular fitness (as a result of physical inactivity).

Related to the idea of whether fat loss is necessary for effective neurocognitive interventions is the notion of the obesity paradox (e.g., Hainer and Aldhoon-Hainerová, 2013; Wang et al., 2016). That is, having a higher BMI actually appears to be protective for some groups. This phenomenon has been observed in older adults aged 73 or older, referred to as the “oldest old” age group. Both cross-sectional and prospective longitudinal studies have reported that there is a positive relationship between BMI and cognitive performance in the oldest old, whereas there is a negative relationship between these two variables in adults ages of 55 and 72 (Luchsinger et al., 2007; Smith et al., 2011; Kim et al., 2016). One possible explanation for this pattern is that lower BMIs in the oldest old may reflect unintentional weight loss due to co-morbid health conditions, rather than healthful lifestyle-related weight maintenance. It's also possible that a reduced BMI could be the *result of* rather than the *cause of* cognitive impairment in this age group (e.g., memory problems could lead to poor nutrition). Individual differences, such as age are therefore complex, yet important, moderators to consider when discussing the effects of obesity on brain and cognition. It's possible that the optimal intervention for promoting and preserving neurocognitive health could differ by age group. For example, increasing cardiovascular fitness and lean muscle may be the critical component for the oldest-old, while other interventions may be just as effective for younger age groups. Regardless, the obesity paradox literature is consistent with the idea that *increased fitness* rather than *decreased fatness* may be the key to successful neurocognitive interventions. In fact, sedentary time and poor cardiovascular health are consistently found to be more predictive of all-cause mortality than obesity (Lee et al., 2010; Sui et al., 2013; Ekelund et al., 2015), a finding that further bolsters this conclusion.

### Limitations and recommendations

Our review should be considered in light of several limitations. First, we chose to focus on interventional evidence to draw our conclusions regarding whether obesity-related neurocogntive outcomes can be reversed via behavioral modifications. In doing so, we chose not to include a detailed summary of the wealth of cross-sectional and correlational evidence that is relevant to this question. However, evidence from interventions in which effects are measured before and after a behavioral modification provide stronger evidence of causal links than correlational or cross-sectional observations. A second limitation of our review is that we are not able to draw precise conclusions regarding the overlap of brain regions showing effects related to obesity, diet, and PA because we did not have access to the data from individual studies. A meta-anlysis on this topic would be useful in order to draw more firm conclusions and to overcome the heterogeneity observed across individual studies.

There are also several limitations in the field that limit more specific conclusions about the associations between obesity, PA, and brain health. One overarching limitation is that it is difficult to separate the role of PA and cardiovascular fitness from the role of body composition when evaluating the effects of weight loss interventions, such as diet. PA levels affect the general health status of an individual and thus may be a confounding variable in all obesity-brain and obesity-cognition relationships reported. On the surface, for instance, the results of dietary interventions for weight loss seem to suggest that increased fitness is not necessary to reverse obesity-related neurocognitive dysfunction. To our knowledge, however, no studies of dietary weight-loss interventions have simultaneously assessed PA levels in the same individuals in order to evaluate whether PA behaviors had also changed. It is therefore not possible to rule out the possibility that PA mediates or moderates the neurocognitive effects observed in these interventions. Future studies of dietary interventions should consider objectively tracking PA levels in order to control for this possibility.

Another limitation of the obesity literature is that many studies use BMI as their main measure of adiposity. However, as mentioned above, BMI is a poor proxy for body composition and it can vary by age and gender. Future studies should therefore make use of more specified methods of measuring body composition (e.g., DEXA) in the future.

Studies of PA are also subject to these same limitations; the majority do not control for adiposity/BMI and peripheral health conditions, such as diabetes and hypertension. This makes separating the contributions of physical activity/fitness from those of weight and body composition difficult, at best. PA interventions in which both activity levels and adiposity measures were collected in the same individuals would help to better disentangle these relationships.

Despite these limitations, this review raises several interesting questions for future research. For instance, would a combined diet and exercise intervention benefit neurocognitive health more than diet or exercise alone? What is the appropriate dose of PA to maximize neurocognitive outcomes? What are the shared mechanisms of both obesity and PA on brain health?

## Conclusions

While PA and obesity share many associations with neurocognitive function, they also differ in several respects. For example, obesity is not merely the instantiation of physical inactivity, nor is physical fitness necessarily indicative of a healthy weight. Understanding the interplay between body composition and physical activity on cognition promotes a more “complex systems” view of cognitive health. In this sense, the brain does not work in isolation of the periphery but instead acts as part of a complex interaction between physiological networks that sustain life. Appreciating how physical health of the entire body impacts brain health is crucial when considering complex public health problems, such as neurocognitive decline. The purpose of this review was to consider together obesity and behavioral interventions as they relate to neurocognitive outcomes in aging. This comparison demonstrated overlap between the neurocognitive mechanisms affected by obesity and those affected by certain behavioral interventions (namely, diet and PA), suggesting that some of the negative neurocognitive consequences of this public health crisis can be reversed. However, our comparison also exposed a need for more rigorous studies directly examining the common and dissociable mediating pathways, cumulative effects, and dose-response relationships in order to fully understand how obesity and promising behavioral interventions, such as PA, affect neurocognitive outcomes. While there are still many unanswered questions, this review highlights that it is important to consider physical health when examining the brain in order to understand how body and brain function act together as a complex system, particularly in the context of aging.

## Author contributions

CS, AW, AM, PG, and KE have seen and approved this manuscript for submission and are accountable for all aspects of the work. CS, AW, AM, PG, and KE made substantial contributions to conceptualizing, drafting, and revising this manuscript.

## Funding

CS is supported by NIH/NIMH T32 MH109986. KE was supported by National Institutes of Health grants R01 DK095172, R01 CA196762, R01 AG053952, P30 MH90333, and P30 AG024827. PG was supported by R01 HL089850. AW was supported by P30 AG024827.

### Conflict of interest statement

The handling Editor declared a past collaboration with one of the authors KE and states that the process nevertheless met the standards of a fair and objective review. The other authors declare that the research was conducted in the absence of any commercial or financial relationships that could be construed as a potential conflict of interest.
